# Pre-treatment optimisation with pulmonary rehabilitation of elderly lung cancer patients with frailty for surgery

**DOI:** 10.1186/s13019-023-02433-9

**Published:** 2023-12-08

**Authors:** Ira Goldsmith, Gemma Chesterfield-Thomas, Hannah Toghill

**Affiliations:** 1 Department of Cardiothoracic Surgery, Morriston Hospital, Swansea, SA6 6NL Wales, UK; 2 Department of Physiotherapy, Morriston Hospital, Swansea, SA6 6NL Wales, UK

**Keywords:** Aging, Pulmonary rehabilitation, Lung cancer surgery, Elderly, Frailty

## Abstract

**Objective:**

Frailty develops as a result of age-related decline in many physiological systems and is associated with increased vulnerability to adverse outcomes following thoracic surgery. We prospectively tested our hypothesis that pre-operative pulmonary rehabilitation (Prehab) improves frailty, as suggested by a frailty index > 3 (FI > 3) and fitness, and thereby reduces the risk of post-surgical complications and death in vulnerable elderly lung cancer patients.

**Methods:**

221 surgical patients, 80 with FI > 3 vs. 141 patients with FI < 3, following Prehab proceeded to surgery. Their Frailty index (FI), dyspnoea scores, performance status (PS), level of activity (LOA) and six-minute walk test (6MWT) prior to and following Prehab were determined. The post-operative length of hospital stay (LOHS), complications, mortality and mid-term survival at 1100 days were compared. Similarly, outcomes for elderly patient ≥ 70 years with FI > 3 (≥ 70,FI > 3) were compared with younger patients < 70 years with FI ≤ 3 (< 70,FI ≤ 3).

**Results:**

Patients with FI > 3 were significantly older, had lower 6MWT and higher thoracoscores hence, 82.5% of patients with FI > 3 vs. 33.3% (p = 0.02) with FI ≤ 3 were considered high risk for surgery and postoperative adverse events. With Prehab there was significant improvement in the FI, dyspnoea scores, PS, LOA and 6MWT. Following surgery, there were no differences in major complication rates (8.8% vs. 9.2% p = ns); LOHS median (IQR) [7 (6.8) vs. 8 (5.5) days]; mortality at 30-days (3.7% vs. 0.7%, p = ns); 90-days (6.3% vs. 2.8%, p = ns) and 1-year survival (81.1% vs. 83.7% p = ns). Survival at 1100 days was (63.2% vs. 71.1%, p = 0.19). Likewise, 87.7% elderly ≥ 70,FI > 3 patients were considered high-risk for surgery and postoperative adverse events vs. 35.1% younger patients < 70,FI ≤ 3 (p = 0.0001). Following Prehab and surgery, there were no significant differences in complications, LOHS, mortality at 365 days between the two groups. Survival at 1100 days for ≥ 70,FI > 3 was 55.2% vs. 79.96% for < 70,FI ≤ 3; (p = 0,01).

**Conclusion:**

Our study suggests that Prehab optimises vulnerable high-risk elderly lung cancer patients with frailty allowing them to undergo surgery with outcomes of post-surgical complications, LOHS and mortality at 365 days no different to patients with no frailty. However, mid-term survival was lower for elderly patients with frailty.

**Supplementary Information:**

The online version contains supplementary material available at 10.1186/s13019-023-02433-9.

## Introduction

Over the past two decades, the number of elderly patients undergoing major surgical procedures have increased substantially. Adverse postoperative outcomes, particularly medical complications, are more frequent in this elderly group of patients when compared with their younger counterparts [1, 2]. Whilst age and pre-existing co-morbidities appear as main predictors of adverse postoperative outcome in this elderly surgical population [3–5], the role of frailty as an independent risk factor for adverse postoperative outcomes, namely, major morbidity, mortality and protracted length of hospital stay (LOHS) and institutional discharge is emerging [6–8].

Frailty develops with aging as a result of age-related decline in many physiological systems [9]. Collectively this may make the elderly more vulnerable to any sudden change in their health status, triggered by minor stressor events. Frailty in elderly patients hence, increases the risk of adverse outcomes, including falls, delirium, disability, and following major surgery to significant morbidity, mortality and a protracted LOHS [9–11]. Hence, the importance of frailty following major surgery is acknowledged in ‘The National Confidential Enquiry into Patient Outcome and Death’ report, which recommends that co-morbidity, disability and frailty be clearly recognised as independent markers of risk in the elderly [8]. At present, an increasing number of people over the age of 65 years are being diagnosed with lung cancer and as a consequence the number of elderly patients undergoing thoracic surgery is increasing [12]. Hence, a comprehensive assessment of elderly patients for co-morbidity, disability and frailty and their vulnerability to increased adverse post-operative outcome is important. Suitable steps can then be taken to improve the identified, modifiable factors and thereby optimise elderly patients with frailty to undergo major lung resection surgery safely.

The benefits of Prehab for patients undergoing lung resection for NSCLC is being increasingly recognized [13–18]. At our institution we have a standardised Prehab protocol, which has been running successfully since 2017 and recently published [13]. Data is collected prospectively to monitor the impact of Prehab on patients being referred for thoracic surgery. Occasionally, the program receives referral of patients for systemic anticancer treatment, radiotherapy or benign conditions. Patients are considered high-risk or inoperable on subjective fitness criteria of Medical Research Council dyspnoea scale > 2, impaired World Health Organisation performance status (PS) > 1, decreased levels of activity (LOA) and frailty index (FI) > 3, and objective functional criteria based on pulmonary function tests namely < 50% forced expiratory volume (FEV1) or < 50% transfer factor for carbon monoxide (DLCO) [13, 19, 20].To help recognise and measure frailty we use the Canadian Study of Health and Aging (CSHA) frailty index. This is a 9-point CSHA questionnaire offering a quick subjective assessment tool in a surgical outpatient setting to detect and grade levels of frailty in the elderly and predict risk for death (Table 1) [13, 21]. On this scale, each level of frailty was ascribed an incremental number to grade the level of frailty as an index (FI). Patients with FI > 3 were considered as having clinically relevant frailty and those ≤ 3 as having no frailty.

**Table 1:** Canadian Study of Health and Aging Clinical Frailty Scale (13,21)

Canadian Study of Health and Aging Clinical Frailty Scale		
Index	Fitness	Definition
1	Very fit	People who are robust, active, energetic and motivated. They tend to exercise regularly and are among the fittest for their age.
2	Fit	People who have no active disease symptoms but are less fit than category 1. Often, they exercise or are very active occasionally.
3	Managing well	People whose medical problems are well controlled, even if occasionally symptomatic, but often are not regularly active beyond routine walking.
4	Very mild frailty	Previously vulnerable, this category marks early transition from complete independence. While not dependent on others for daily help, often symptoms limit activities e.g., ‘slowed up’ and/or being tired during the day.
5	Mild frailty	People who often have more evident slowing, and need help with high order instrumental activities of daily living. Typically mild frailty impairs shopping and walking outside alone, meal preparation, medications and begins to restrict light housework.
6	Moderate frailty	People who need help with all outside activities and with keeping house. Inside, they often have problems with stairs and need help with bathing and might need minimal assistance with dressing.
7	Severe frailty	Completely dependent for personal care, from whatever cause (physical or cognitive). They seem stable and not at high risk of dying (within 6-months).
8	Very severe frailty	Completely dependent for personal care and approaching end of life. Typically they could not recover even from a minor illness

The aim of the study was to review our prospectively collected data and determine whether Prehab, by improving frailty along with the subjective fitness criteria, makes elderly surgical patients with frailty, as suggested by a frailty index > 3, suitable for surgery and thereby allows them to safely undergo curative lung resection with outcomes, namely post-operative complications, LOHS, mortality and survival following surgery no different to patients with no frailty, as suggested by a frailty index of ≤ 3.

## Materials and methods

Our pragmatic study was carried out between January 2017 and December 2019 at Morriston Hospital, Swansea, United Kingdom [13]. The service received 434 referrals for pre-treatment optimization of patients with Prehab from the lung cancer Multi-Disciplinary Teams (MDTs) across South West Wales. The referral criteria were either/and > 1 dyspnoea; >1 PS; age > 70 years; FI > 3; borderline or poor pulmonary function (< 50% FEV1 or DLCO); patients currently smoking; sedentary patients despite having adequate FEV1 or DLCO; or on clinical decision made by the treating physician who deemed that the patient would benefit from Prehab prior to treatment, for example, for psychological or other reasons.

### Patients excluded

10.4% (n = 45) of patients diagnosed with other cancers or benign diseases were excluded from the study as were 17.2% (n = 75) of patients who either declined, did not attend, had high cardiovascular risk for Prehab, died prior to Prehab or proceeded straight to surgery.

### Participants

The diagnosis and stage of lung cancer of the remaining 314 patients were validated. For clinical lung cancer staging the 8th edition of the International Association for the Study of Lung Cancer staging system was used [22]. Assessment of patients and data collection prior to commencing Prehab and on completion of Prehab were carried out by our cardiothoracic physiotherapists. Assessment for frailty was carried out using the CSHA Clinical Frailty Scale at first assessment and on completion of Prehab (Table 1) [13, 21]. Patients with FI > 3 were considered as having clinically relevant frailty. Assessment for risk of death was carried out using the thoracoscore, which is the recommended scoring system for in-hospital death [19, 23]. Different patients had different rehabilitation requirements, and as patients demonstrated improvement in their dyspnoea score, PS and LOA, thus meeting the subjective fitness criteria for surgery, proceeded to surgery. Those with a greater risk of cancer progression, due to a long waiting time on the lung cancer pathway or N1 or N2 nodal disease, proceeded to treatment without completing their final assessment.

Following surgery, outcomes measured were major complications, LOHS, mortality and survival at 365 and 1100 days. The extended Clavien-Dindo classification for grading complications was used and grades 3a to 5 considered major complications [13]. Adverse Cardio-respiratory events, a more relevant outcome measure for Prehab, were additionally assessed. Patients were discharge from hospital following removal of intercostal drain(s) and meeting standard discharging criteria of asepsis, adequate pain control, normal bowel movement, ability to carry out activities of daily living and having successfully climbed a flight of stairs (13 steps) with the physiotherapists.

### Study

To test our hypothesis and determine the impact of Prehab on patients with frailty we initially determined the impact of Prehab in the total cohort. Patients with FI > 3 were first compared with patients with FI ≤ 3. Next, to test the hypothesis and determine the impact of Prehab specifically on elderly patients with frailty for surgery, patients with stage III and IV disease requiring radiotherapy, chemotherapy and palliative care were excluded from the analysis. Elderly patients ≥ 70 years with FI > 3 (≥ 70,FI > 3) being considered for surgery, were compared with younger patients < 70 years with FI ≤ 3 (< 70,FI ≤ 3) for surgery. The percentage of patients unfit to proceed with surgery at first assessment and the percentage of patients suitable to proceed with surgery following Prehab were determined.

### Pre-operative pulmonary rehabilitation protocol

Our Prehab program, delivered by trained cardiothoracic physiotherapists, are describe in supplementary table and previously, based on standard guidelines [13, 24, 25]. Prehab was provided over 2–4 weeks with supervised two weekly sessions of 70 min each at our Prehab centre or outreach units, along with exercises patients could carry out at home three times daily.

### Statistical analysis

Descriptive statistics are expressed as percentage (numbers), and means and 95% confidence interval (± 95% CI) or median and interquartile range (IQR). Intergroup differences were analysed by 2-sided unpaired t-test, Mann–Whitney test, chi-square test or the Fisher’s exact test as appropriate. Due to missing data, as some patients had to proceed to surgery quickly or patients exercising at home who could not travel back for reassessment, we were unable to use the paired samples t-test or ANOVA. The Kaplan Meier log rank test was used to estimate survival at 365 days and 1100 days following surgery. Statistical analysis of the data was performed using IBM SPSS Statistics, 2018 software. We used the Strengthening the Reporting of Observational Studies in Epidemiology guidelines for reporting our pragmatic study.

## Results

### Baseline results for the whole cohort

The baseline characteristics of the total cohort of 314 patients are described in Table 2. Of the 137 patients identified with FI > 3 and considered vulnerable to adverse events, 25.2% (79) had FI of 4; 12.4% (39) FI of 5; 5.7% (18) FI of 6 and 0.3% (1) FI 7.

**Table 2:** Baseline patients’ characteristics of 314 patients with lung cancer for Prehab

Parameter	Descriptor	n	%
**Gender**	**Male** **Female**	155 159	49.4 50.6
**Age in years**	**Mean (± 95% CI)**	71.8 ± 0.9	
**Referral to assessment days**	**Mean (± 95% CI)**	14.4 ± 1.2	
**Stage of lung cancer**	**IA1** **IA2** **IA3** **IB** **IIA** **IIB** **IIIA** **IIIB** **IVA**	31 49 38 74 13 34 53 14 8	9.9 15.6 12.1 23.6 4.1 10.8 16.8 4.6 2.5
**Dyspnoea score**	**0–2** **3–4**	150 160	48.4 51.6
**Performance status**	**0–1** **2–4**	163 147	52.6 47.4
**Level of activity**	**Sedentary** **Moderately active** **Very active**	207 77 27	66.6 24.7 8.7
**Frailty**	**1–3** **4–9 (venerable for adverse events)**	172 137	55.7 44.3
**Smoking habit**	**Current smoker** **Ex- smoker** **Never smoked**	61 217 35	19.5 69.3 11.2
**Smoking cessation advice**	**Accepted** **Declined** **None**	43 9 8	71.7 15.0 13.3
**FEV1 (litres)**	**Mean (± 95% CI)**	1.77 ± 0.07	
**FEV1 (% predicted)**	**Mean (± 95% CI)**	77.01 ± 2.8	
**DLCO (% predicted)**	**Mean (± 95% CI)**	66.2 ± 2.2	
**Thoracoscore (%)**	**Mean (± 95% CI)**	2.7 ± 0.3	
**Six minute walk test (mts)**	**Mean (± 95% CI)**	306.4 ± 15.4	
**Not fit for**	**Surgery** **Any radical treatment**	194 185	61.9 58.9
**Assessment to surgery days**	**Mean (± 95% CI)**	44.6 ± 6.9	
**Sessions of Pre-hab**	**Mean (± 95% CI)**	3.4 ± 0.5	

*Values are numbers (percentage) unless otherwise stated. CI = confidence interval. Level of activity was measured using the Borg scale and assessments recorded as sedentary, moderately active or active.*

**Fig. 1:**
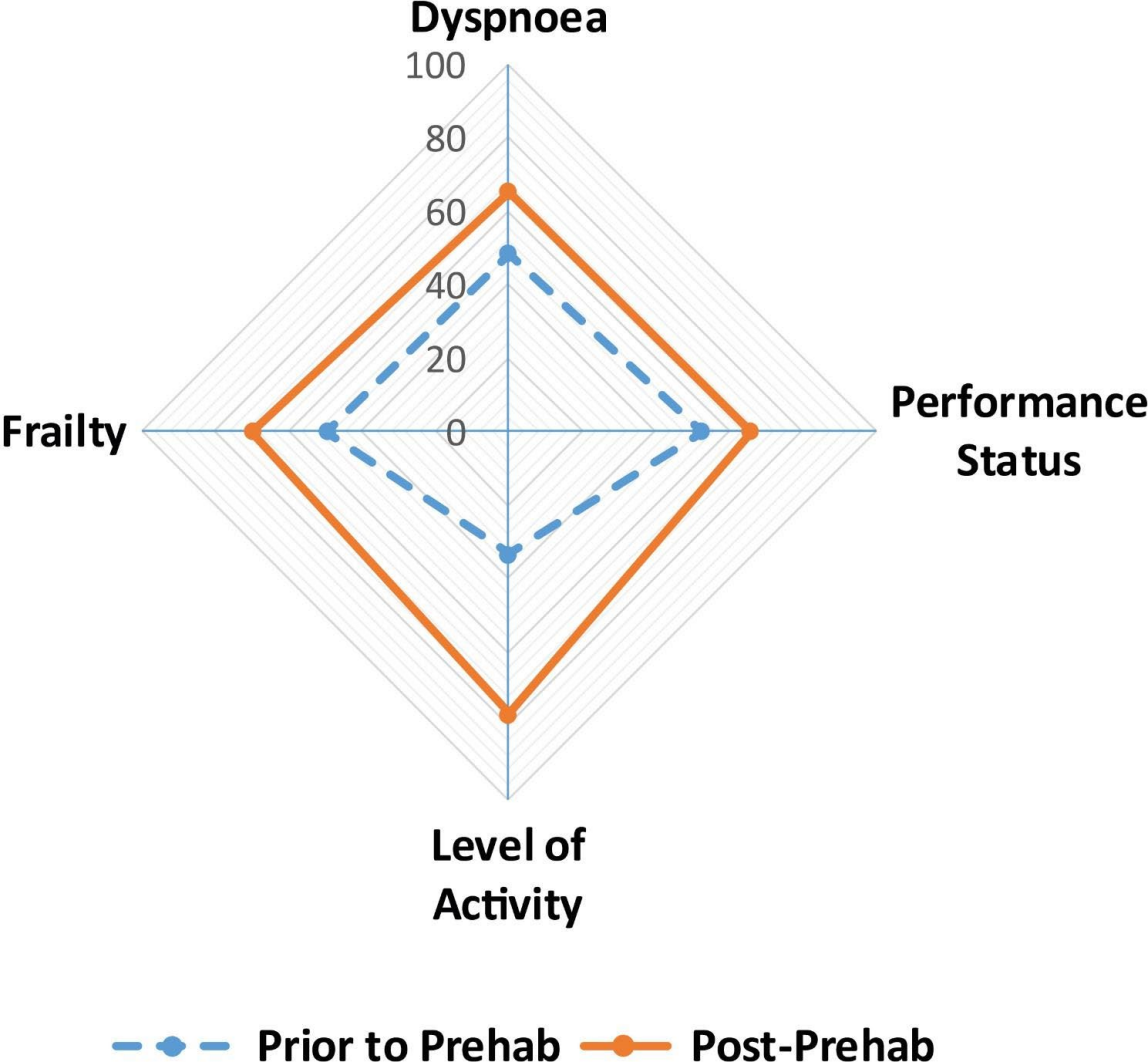
Impact of Prehab. The spider plot graphically describes the significant, overall improvement in the fitness of patients achieved with Prehab. There was significant improvement in the frailty index, dyspnoea scores, performance status and levels of activity in the cohort. *Values are percentages prior to and following Prehab*

Impact of Prehab on the total patient cohort: Over an average of 44.6 ± 6.9 days there was a significant improvement in the overall fitness of patients with Prehab as demonstrated graphically in Fig. 1 and in the mean 6MWT distance prior to and following Prehab [306.4 ± 15.4 m vs. 377 ± 26.4 m, (p = 0.00001)] respectively.

At the time of assessment, 58.9% of patients were considered high-risk for any radical treatment (surgery or radiotherapy) (Table 2). Following Prehab 94.6% vs. 58.9% (p < 0.00001) were ready to go forward for radical treatment. Similarly, prior to Prehab 61.9% of patients were not considered suitable to proceed with safe surgery. Following Prehab there was a significant increase, from 38 to 78.2% (p = 0.00001) patients now fit to proceed with surgery. 70.4% (n = 221) of patients proceeded to surgery and a majority (76%) underwent an anatomical lung resection (Table 3). Conversely, 11.1% (n = 35) of patients proceeded to radiotherapy, 9.9% (31) to systemic anti-cancer treatment, 4.5% (14) declined treatment, 0.6% (2) died before any treatment and 3.5% (11) received palliative care, and were excluded from further analysis.

**Table 3:** Outcomes for the 221 patients with lung cancer being considered for surgery following Prehab

Parameter	Descriptor	n	%
**Surgery**	**Lobectomy** **Pneumonectomy** **Segmentectomy** **Wedge resection** **Endobronchial excision** **Failed trial of OLV** **Thoracotomy (open and close)**	140 3 25 45 3 1 4	63.3 1.4 11.3 20.4 1.4 0.5 1.8
**Surgical approach**	**Endobronchial excision** **VATS** **Hybrid VATS** **Thoracotomy** **Failed OLV**	3 24 108 85 1	1.4 10.6 48.9 38.5 0.5
**Cardiorespiratory adverse events**	**Cardiac**: **Atrial fibrillation** **Major cardiac events** **Hypotension/hypertension** **Respiratory**: **Air leak** **Lobar collapse/consolidation** **Respiratory infection/retained secretions** **Pleural effusion/excessive drainage** **Pneumothorax/Surgical emphysema (mild)**	18 3 2 6 8 29 4 10	8.1 1.4 0.9 2.7 3.6 13.1 1.8 4.5
**Post-operative Clavien-Dindo Grade of complications for each patient**	**None** **Minor: 1** **2** **Major: 3a and 3b** **4a** **5**	111 49 41 13 5 2	50.2 22.2 18.5 5.9 2.3 0.9
**Step up care**		10	4.5
**Length of stay**	**HDU days [median (IQR)]** **Ward days [median (IQR)]** **Hospital days [median (IQR)]**	3 (2) 3 (3) 7 (6)	
**Mortality**	**In-hospital** **30-day** **90-day** **1 year**	2 4 10 37	0.9 1.8 4.5 16.7
**Survival**	**365 days (CI)** **1100 days (CI)**	83.3% 60.04%	78.0-87.9 58.5–72.5

*Values are numbers (percentage) unless otherwise stated. IQR = Interquartile range; CI = confidence interval.*

Post-surgical outcomes are described in Table 3 for the 221 patients for surgery. Following surgery, cardiac dysrhythmia occurred in 8.1% of patients, a major cardiac event in 1.36% of patients and 25.7% developed respiratory complications.

### Comparison of surgical patients with frailty vs. those with no frailty

**(i) Entire surgical cohort of 221 patients**: The mean age of patients with FI > 3 was significantly higher at 72.9 ± 1.6 years vs. 70.9 ± 1.3 years (p < 0.034) for patients with FI ≤ 3 (Table 4).

**Table 4:** Baseline characteristics of patients with no frailty compared with patients with frailty

Parameter		FI ≤ 3 vs. FI > 3			< 70,FI ≤ 3 vs. ≥70,FI > 3		
		**FI ≤ 3** (n = 141)	**FI > 3** (n = 80)	**P**	**< 70,FI ≤ 3** (n = 57)	**≥ 70,FI > 3** (n = 57)	**p**
**Gender**	**M:F**	69:72	36:44	ns	27:30	28:29	ns
**Age (years)**	**Mean (**± 95%CI**)**	72.9 ± 1.6	70.9 ± 1.3	0.034	63. 9 ± 1.2	76.7 ± 1.1	< 0.00001
**Stage of lung cancer**	**I** **II** **III** **IV**	93 (66) 22 (15.6) 25 (17.7) 1 (0.7)	56 (70) 12 (15) 12 (15) -	ns	19 (33.3) 28 (49.1) 10 (17.5) -	44 (77.2) 8 (14.1) 5 (8.8)	ns
**Performance status**	**0–1** **2–4**	114 (82) 25 (18)	16 (41.2) 64 (58.8)	0.00001	43 (78.2) 12 (21.8)	13 (22.8) 44 (77.2)	< 0.00001
**Dyspnoea score**	**0–2** **3–4**	101 (72.7) 38 (27.3)	19 (23.7) 61 (76.3)	0.00001	36 (65.5) 19 (34.5)	11 (19.3) 46 (80.7)	< 0.00001
**Level of activity**	**Sedentary** **Active**	57 (40.7) 83 (59.2)	77 (96.3) 3 (3.7)	0.00001	25 (44.6) 31 (55.4)	55 (96.5) 2 (3.5)	< 0.00001
**Smoking habit**	**Current smoker** **Previous smokers**	20 (14.2) 102 (72.3) 19 (13.5)	21(26.3) 51 (63.75) 8 (10)	0.07	14 (24.6) 36 (63.1) 7 (12.3)	8 (14.0) 42 (73.7) 7 (12.3)	ns
**Smoking cessation advice**	**Yes** **Declined** **No**	14 (66.7) 5 (23.8) 2 (9.5)	16 (84.2) 2 (10.5) 1 (5.3)	ns	11 (78.6) 2 (14.3) 1 (7.1)	4 (50) 2 (25) 2 (25)	ns
**FEV1 (litres)**	**Mean (**± 95%CI**)**	1.84 ± 0.11	1.81 ± 0.37	ns	1.86 ± 0.2	1.79 ± 0.2	ns
**FEV1 (% predicted)**	**Mean (**± 95%CI**)**	79.85 ± 4.2	82.81 ± 4.8	ns	72.4 ± 6.6	83.8 ± 6.1	0.007
**DLCO (% predicted)**	**Mean (**± 95%CI**)**	71.9 ± 3.1	63.46 ± 3.7	0.0005	68.94 ± 4.6	63.42 ± 4.7	0.05
**Thoracoscore (%)**	**Mean (**± 95%CI**)**	2.1 ± 0.3	3.33 ± 0.5	0.00002	1.78 ± 0.3	3.49 ± 0.7	< 0.00001
**6MWT (mts)**	**Mean (**± 95%CI**)**	378.11 ± 17.7	218.68 ± 24.1	0.00001	399.69 ± 29.6	211.82 ± 26.3	< 0.00001
**Sessions of Prehab**	**Mean (**± 95%CI**)**	3.7 ± 0.7	3.6 ± 1.1	ns	3.31 ± 0.9	3.24 ± 1.4	ns
**Assessment to surgery days**	**Mean (**± 95%CI**)**	44.06 ± 8.5	62.44 ± 18.7	0.02	37.42 ± 12.5	56.6 ± 16.0	0.04
**Considered not fit for**	**Surgery** **Radical treatment**	47 (33.3) 45 (31.9)	66 (82.5) 27 (36.9)	0.00001 ns	20 (35.1) 18 (31.6)	50 (87.7) 11 (26.2)	< 0.00001 0.0066

*Values are numbers (percentage) unless otherwise stated. CI = confidence interval.*

There were no differences in the gender, stage of lung cancer or referral to assessment days between the two groups. A significantly higher percentage of patients with FI > 3 had poor PS scores, dyspnoea scores, led a sedentary life style, and had significantly lower DLCO, 6MWT distance and high thoracoscores compared with patients with FI ≤ 3, each suggesting higher risk for post-operative adverse events and mortality for patients with FI > 3 (Table 4). Hence, a significantly higher proportion of patients with a FI > 3 were considered not ready to proceed with surgery compared with patients with a FI ≤ 3.

Following their assessment for Prehab, a similar proportion of patients in both the groups received smoking cessation advice (Table 4). The duration of Prehab was significantly longer at 62.4 ± 18.7 days for patients with FI > 3 compared to 44.06 ± 8.5 days (p < 0.02) for patients with FI ≤ 3. With Prehab there was clinical and statistically significant improvement in fitness of patients with FI > 3 (Table 5) and following Prehab 95.2% of patients with FI > 3 were considered fit to proceed with surgery. There were no differences in the surgical approach or surgical resection in both groups or in the outcomes following surgery (Table 6).

**Table 5:** Optimization of high risk patients with Frailty Index > 3 with Prehab

Parameter		FI > 3			≥ 70,FI > 3		
		Prior to Prehab n (%)	Following Prehab n (%)	p	Prior to Prehab n (%)	Following Prehab n (%)	p
**Performance status**	**0–1** **2–4**	16 (20) 64 (80)	21 (41.2) 30 (58.8)	0.008	13 (22.8) 44 (77.2)	12 (35.3) 22 (64.7)	0.3
**Dyspnoea score**	**0–2** **> 2**	19 (23.7) 61 (76.3)	29 (56.8) 22 (43.2)	0.0001	11 (19.3) 46 (80.7)	17 (50) 17 (50)	0.005
**Level of activity**	**Sedentary** **Active**	77 (96.3) 3 (3.7)	20 (40) 30 (60)	0.00001	55 (96.5) 2 (3.5)	14 (42.4) 19 (57.6)	< 0.0001
**Frailty**	**1–3** **4–9**	0 (0) 80 (100)	20 (40) 30 (60)	0.00001	0 57 (100)	11 (33.3) 22 (66.7)	0.002
**6MWT**	**(Mean** ± **95% CI)**	218.68 ± 24.2	306.54 ± 75.7	0.04	211.8 ± 26.3	248.1 ± 73.6	ns
**Considered not fit for**	**Surgery** **Radical treatment**	66 (82.5) 27 (36.9)	3 (4.8) 2 (3.2)	0.00001 0.00001	50 (87.7) 11 (26.2)	2 (4.6) 2 (4.6)	< 0.00001 < 0.00001

*Values are numbers (percentage) unless otherwise stated. CI = confidence interval.*

**Table 6:** Outcomes following Prehab and surgery in low risk patients with no Frailty compared with the high risk patients with Frailty

Parameter		FI ≤ 3 vs. FI > 3			< 70,FI ≤ 3 vs. ≥70,FI > 3		
		**FI ≤ 3** (n = 141)	**FI > 3** (n = 80)	**P**	**< 70,FI ≤ 3** (n = 57)	**≥ 70,FI > 3** (n = 57)	**p**
**Type of surgery**	**Lobectomy** **Segmentectomy** **Pneumonectomy** **Wedge resection** **Endobronchial excision** **Thoracotomy** **Failed OLV trial**	98 (69.5) 15 (10.6) 3 (2.1) 24 (17.0) 1 (0.7)	42 (52.5) 11 (13.8) - 21 (26.3) 2 (2.5) 3 (3.7) 1 (1.2)	0.078	39 (68.4) 5 (8.8) 1 (1.8) 11 (19.3) 1 (1.8)	31 (55.4) 7 (12.5) - 14 (25) 2 (3.6) 1 (1.8) 1 (1.8)	ns
**Surgical approach**	**Minimally invasive** **Thoracotomy** **Failed OLV trial**	88 (62.4) 53 (37.6)	47 (58.8) 32 (40) 1 (1.2)	ns	35 (61.4) 22 (38.6)	35 (45.6) 21 (36.8) 1 (1.8)	ns
**HDU**	**Median (IQR)**	4 (2)	3 (3)	ns	3 (3)	3 (3)	ns
**Ward**	**Median (IQR)**	3 (3.7)	3 (3)	ns	3 (4)	3 (3)	ns
**LOHS**	**Median (IQR)**	8 (5.5)	7 (6.8)	ns	8 (7)	7 (6.5)	ns
**Complications**	**Major**	13 (9.2)	7 (8.8)	ns	8 (14.0)	6 (10.5)	ns
**Cardio-respiratory events**	**Cardiac**: **Atrial fibrillation** **Major cardiac events** **Hypotension/hypertension** **Respiratory**: **Air leak** **Lobar collapse/consolidation** **Respiratory infection/retained secretions** **Pleural effusion/excessive drainage** **Pneumothorax/Surgical emphysema (mild)**	13 (19) 1 (1.4) 1 (1.4) 5 (7.1) 3 (4.3) 17 (24.3) 4 (5.7) 6 (8.6)	5 (12.2) 2 (4.8) 1 (2.4) 1 (2.4) 5 (12.2) 12 (29.3) - 4 (9.7)	ns ns	4 (7.0) 0 1 (1.8) 2 (3.5) 2 (3.5) 11 (19.3) 1 (1.7) 3 (5.3)	5 (8.8) 1 (1.8) 2 (3.5) 0 2 (3.5) 11 (19.3) 0 2 (3.5)	ns ns
**Recidivism**		8 (5.7)	2 (2.5)	ns	4 (7.0)	2 (3.5)	ns
**Post-operative Mortality**	**In-hospital**	1 (0.7)	1 (1.2)	ns	1 (1.8)	(1 (1.8)	
	**30 days**	1 (0.7)	3 (3.7)	ns	1 (1.8)	2 (3.5)	ns
	**90 days**	4 (2.8)	5 (6.3)	ns	3 (5.3)	3 (5.3)	ns
	**1 year**	21 (14.9)	16 (20)	ns	8 (14.0)	12 (21)	ns
**% Survival (range)**	**365 days (CI)** **1100 days (CI)**	85.0 (75.1–1.2) 71.1 (58.2–80.7)	80.0 (72.4–85.7) 63.2 (53.6–71.4)	ns ns	85.96 (73.9–92.7) 79.96 (66.9–88.4)	76.8 (63.7–85.9) 55.2 (39.9–68.1)	ns 0.01

*Values are numbers (percentage) unless otherwise stated. IQR = Interquartile range; CI = confidence interval.*

**(ii) Elderly patients ≥ 70 years with frailty vs. younger patients < 70 years with no frailty**: The mean age of patients ≥ 70,FI > 3 was significantly higher than patients < 70,FI ≤ 3 (Table 4). However, there were no differences in the gender, stage of lung cancer, smoking habits or sessions of Prehab delivered between the two groups (Table 4). A significantly higher percentage of elderly patients ≥ 70,FI > 3 had poor PS, dyspnoea scores, led a sedentary life style, had lower predicted FEV_1_ and DLCO, 6-MWT distance and high thoracoscores compared with younger patients < 70,FI ≤ 3, and required a longer period of optimisation with Prehab (Table 4).

With Prehab there was clinical and statistically significant improvement in PS, dyspnoea, LOA, and FI hence, fitness of patients ≥ 70,FI > 3 (Table 5). Hence, following Prehab 95.4% of elderly patients ≥ 70,FI > 3 were considered fit to proceed with surgery (Table 5). At surgery, there were no differences in the surgical approach or surgical resection in both the groups (Table 6) and following surgery in their outcomes (Table 6). On follow-up, the mean follow-up days for < 70,FI ≤ 3 was 804.2 days vs. 960.3 days for those ≥ 70,FI > 3, (p = 0.04). The Kaplan Meier survival at 1100 days were significantly higher for younger patients with no frailty at 79.96% compared to 55.2% (p < 0.01) for significantly older patients with frailty.

## Discussion

Our study is unique in that we have used the 9-point Clinical Frailty Scale as an index to assess patients for levels of frailty [21, 26]. The study demonstrates that 44.3% (137/314) of patients with lung cancer referred for Prehab in general, and 36% (n = 80/221) of lung cancer patients for surgery have frailty, which is consistent with published literature (8–10). Hence, our study suggests that a third of patients for surgery requiring Prehab are vulnerable to adverse postoperative complications, a protracted LOHS and mortality. This is in keeping with their corresponding poor dyspnoea scores, PS scores, low activity levels, significantly lower 6-MWT, DLCO and high thoracoscores at assessment for Prehab/surgery, and when compared with patients having no frailty [7, 13, 19, 20]. By providing a standardised Prehab program and improving their FI and fitness, our study shows that it is feasible to optimise elderly patients with frailty, who are considered either high risk for surgery or inoperable [27]. The duration of Prehab required is, however, significantly longer for this group of patients. Following optimisation with Prehab, our study shows that elderly patients with frailty are able to proceed with surgery with outcomes similar to patients with no frailty and considered to have low risks for adverse events or death [13, 19].

Our study takes into account that 90% of patients with FI > 3 were ever-smokers hence, likely to have underlying smoking related cardiopulmonary disease. Loss of lung tissue with surgical resection in such patients may grossly impair their post-operative ventilatory function or diffusion capability, predisposing them to dyspnoea, complications and death [19,26,28]. Hence, a comprehensive assessment of elderly patients for co-morbidity and modifiable factors is important. Our study demonstrates that with a standardised Prehab program, it is possible to improve their modifiable factors of dyspnoea, PS, LOA, FI and achieve optimisation for surgical resection with good outcomes [13, 14, 28].

The five-year survival of patient with lung cancer amongst other cancers is poor [29] and a multi-disciplinary approach is required to improve the survival of patients with lung cancer. We believe that by offering standardised Prehab to vulnerable elderly lung cancer patients with resectable disease and frailty, is one amongst other strategies that will help improve resection rates, especially as the outcomes are similar to low risk patients. Increasing the numbers of patients undergoing surgery with curative intent may contribute to improving the overall long-term survival of lung cancer patients.

Interestingly our study, in keeping with the findings of a randomized trial by Wolfram Karenovics et al. [30], shows that there were no differences in the one-year survival of patients with FI > 3 compared with patients with FI ≤ 3. However, the long-term actuarial survival at 1100 days was significantly higher for younger patients < 70 years with FI ≤ 3 compared with elderly patients ≥ 70 years with FI > 3. In our study, the follow-up days were significantly longer in the elderly group. This and an older age over time may have negatively impacted upon the long term survival of elderly patients ≥ 70 year with FI > 3, which a randomized study may help confirm.

Our study has certain limitations. This is a single-institution, pragmatic, real life provision of a standardized Prehab program to optimise lung cancer patients with frailty for surgery and may not fully address confounding factors. Objectively counting a patient’s clinical deficits for frailty, which although reproducible and correlates highly with mortality, is unwieldy [30]. Nevertheless, a randomized setting would allow such objective comparison with a control population and address confounding factors. The program is limited in its provision of service to a group of selected patients requiring optimisation prior to surgical treatment. The study is also limited in its ability to routinely re-check pulmonary functions, especially FEV1, DLCO, and cardiopulmonary exercise test (CPEX) following Prehab [19, 20, 30]. Only a small number of patients underwent CPEX testing, which was not routinely carried out. With significant improvements observed with Prehab on frailty, dyspnoea, PS, LOA and 6MWT, patients were able to proceed to surgery without requiring routine CPEX testing. The impact on economic benefits and on the quality of life of patients were not assessed, nor the disease free survival.

In conclusion, our pragmatic study demonstrates that with a standardised Prehab program, vulnerable high-risk elderly patients with high frailty index for lung resection, can be suitably optimised to proceed to safe surgical resection and have outcomes similar to low-risk patients with low frailty index. Therefore, at lung cancer MDTs the management plan for high-risk elderly patients with frailty, who are otherwise deemed vulnerable to adverse events and death, should include consideration of referral for a period of structured Prehab. Bearing in mind that optimisation with Prehab takes longer in this elderly group of patients, the duration of Prehab is guided by their stage of disease and risk of progression. Those suitably optimised with Prehab can be expected to safely proceed with curative lung resection. Nevertheless, a suitably powered randomized control trial is required to confirm our observations and establish whether a structured Prehab program helps improve the long-term survival of elderly lung cancer patients with frailty.

### Electronic supplementary material

Below is the link to the electronic supplementary material.

Supplementary Material 1

## Data Availability

Access to individual-level data is governed by and processed under the United Kingdom’s Data Protection Act (DPA) 2018, the EU General Data Protection Regulation (GDPR) and the Common Law Duty of Confidentiality (CLDC). Researchers may obtain the relevant approval and access data via the Swansea Bay University Health Board.
