# Supplementary material for: Pre-treatment optimisation with pulmonary rehabilitation of elderly lung cancer patients with frailty for surgery
Source: J Cardiothorac Surg. 2023 Dec 8;18:356. doi: 10.1186/s13019-023-02433-9 (PMC10704745; doi:10.1186/s13019-023-02433-9)
Supplement: Supplementary file 1 — Supplementary Material 1 [file 13019_2023_2433_MOESM1_ESM.pdf]

Supplementary 1

Pre-Operative Pulmonary Rehabilitation Protocol (13).

Elements	Description of exercise
**Respiratory muscle training and breathing exercises:**	Strengthening the patients muscles of respiration techniques for muscle relaxation and control; improving their diaphragmatic breathing and control; coordinating their breathing process; and re-training their respiratory muscles with the use of incentive spirometry performed three times daily for the duration of Prehab. Effective airway clearance techniques were taught and performed three times daily..
**Cardiovascular exercises:**	Stationary cycle ergometry and walking exercise training whilst monitoring their heart rate, blood pressure and oxygen saturations performed twice weekly. Borg scale used twice at every session to measure the intensity at which each patient was working and to guide them to increase or decrease their effort as required. Twice daily walking exercises and stair climbing exercises at home and shoulder movement exercises, movements of upper and lower limb joints, standing up from a sitting position and walking to facilitate early post-operative ambulation and activities of daily living.
**Education:**	Health education and smoking cessation advice to all current smokers and maintained after surgery.
**Pharmacology agents:**	When necessary nicotine replacement therapy to quit smoking and bronchodilator therapy if required.
